# Withaferin A: a potential therapeutic agent against COVID-19 infection

**DOI:** 10.1186/s13048-020-00684-x

**Published:** 2020-07-19

**Authors:** Alex R. Straughn, Sham S. Kakar

**Affiliations:** 1 James Graham Brown Cancer Center, University of Louisville, Louisville, KY 40202 USA; 2 Department of Physiology, University of Louisville School of Medicine, 500 South Floyd Street, Louisville, KY 40202 USA

**Keywords:** Coronavirus, Pandemic, Withaferin a, Withanolides, Ashwagandha, Cancer

## Abstract

The outbreak and continued spread of the novel coronavirus disease 2019 (COVID-19) is a preeminent global health threat that has resulted in the infection of over 11.5 million people worldwide. In addition, the pandemic has claimed the lives of over 530,000 people worldwide. Age and the presence of underlying comorbid conditions have been found to be key determinants of patient mortality. One such comorbidity is the presence of an oncological malignancy, with cancer patients exhibiting an approximate two-fold increase in mortality rate. Due to a lack of data, no consensus has been reached about the best practices for the diagnosis and treatment of cancer patients. Interestingly, two independent research groups have discovered that Withaferin A (WFA), a steroidal lactone with anti-inflammatory and anti-tumorigenic properties, may bind to the viral spike (S-) protein of SARS-CoV-2. Further, preliminary data from our research group has demonstrated that WFA does not alter expression of ACE2 in the lungs of tumor-bearing female mice. Downregulation of ACE2 has recently been demonstrated to increase the severity of COVID-19. Therefore, WFA demonstrates real potential as a therapeutic agent to treat or prevent the spread of COVID-19 due to the reported interference in viral S-protein to host receptor binding and its lack of effect on ACE2 expression in the lungs.

## Introduction

The novel coronavirus disease 2019 (COVID-19) has rapidly spread around the world since it was first reported in December 2019 within Wuhan, China as a pneumonia of unknown etiology [1]. Severe acute respiratory syndrome coronavirus-2 (SARS-CoV-2), termed by the World Health Organization (WHO), represents the third large-scale epidemic related to coronaviruses [1]. Although the disease was first reported within China, a retrospective study has subsequently found evidence that SARS-CoV-2 was spreading within France 4 days before it was first reported in Wuhan, China and 1 month before the first official case in the country [2]. Since its initial discovery, SARS-CoV-2 has spread worldwide, infecting over 11.5 million people and led to the death of more than 530,000 people as of July 6th, 2020 [3]. The severity of the disease widely ranges from an asymptomatic disease-state to patients exhibiting acute respiratory distress syndrome (ARDS), necessitating critical medical intervention to attempt to prevent patient death [4]. It was subsequently discovered that Angiotensin-converting enzyme 2 (ACE2) is a functional receptor for the SARS-CoV-2 spike (S-) protein, allowing the virus to enter cells [5]. ACE2 is a potent negative regulator of the renin angiotensin system (RAS), which is critical for maintaining the homeostasis of RAS.

The ACE2 gene is composed of 805 amino acids and is a type I integral membrane glycoprotein. ACE2 degrades angiotensin (Ang)-II, a potent vasoconstrictor (that is also pro-inflammatory and promotes fibrosis), and converts it into Ang (1–7) [6]. Ang (1–7) is a vasodilator, that also inhibits proliferation and apoptosis [6]. Beside the systemic effect on blood pressure regulation, ACE2 has local regulatory effects in the pathological changes of several organs, including the heart, kidney, and lungs [7]. ACE2 is highly expressed in lung alveolar cells, providing the main entry site for the virus into human host [8]. In addition to expression of ACE2 in lung alveolar cells, it is also expressed in various tissues, including: the vascular system (endothelial cells, migratory angiogenic cells and vascular smooth muscle cells), heart (cardiofibroblasts, cardiomyocytes, endothelial cells, pericytes, and epicardial adipose cells) and kidneys (glomerular endothelial cells, podocytes and proximal tubule epithelial cells), liver (cholangiocytes and hepatocytes), retina (pigmented epithelial cells, rod and cone photoreceptor cells, and Müller glial cells), enterocytes of the intestines, circumventricular organs of the central nervous system, and the upper airway (goblet and ciliated epithelial cells) [9].

There are two subunits of the SARS-CoV-2 S-protein: the S1 subunit has a receptor binding domain that engages with the host cell receptor ACE2, and the S2 subunit is involved in regulating fusion between the viral and the host membrane [10]. It has been reported that SARS-CoV-2 has a ten times higher affinity to ACE2 compared to SARS-CoV, which is consistent with the higher efficiency of infection of SARS-CoV-2 [11]. While no cure has currently been found, several clinical trials are being performed to determine what the most efficacious treatment regimen is for COVID-19, with an extensive list of potential therapies detailed in a review by Gosain et al. [12]. Currently, patient management involves supportive treatment and measures to prevent further spread of the virus [13]. Despite differences in patient population characteristics between Europe and China, two of the main determinants of patient mortality risk that were found in both groups are age and the presence of underlying comorbid conditions [14, 15]. One such underlying condition associated with an increase in COVID-19 patient mortality is the presence of cancer [16].

### Cancer patients and the COVID-19 epidemic

Due to their potentially immune-compromised status, the proper treatment of cancer patients is a real and serious problem being faced by oncologists, regardless of if the patient is experiencing a SARS-CoV-2 infection [16]. Data from four SARS-CoV-2 hot spots (the United States, Italy, Spain and China) has shown that cancer patients infected with the novel coronavirus have a significantly increased risk of admission to an intensive care unit (ICU) and/or requiring mechanical ventilation, as well as an increase in patient mortality [15, 17–19]. In a retrospective study, the fatality rate for cancer patients in China infected with COVID-19 was found to be approximately 28% [20], compared to the overall symptomatic fatality rate of 1.4% or the crude mortality rate of 4.5% in China [21]. Perhaps unsurprisingly, the fatality rate of lung cancer patients with SARS-CoV-2 has been fairly grim, with a New York cohort study exhibiting a 55% fatality rate [19]. Cancer patients and their oncologists are currently facing the dilemma as to whether or not the patient should begin or continue therapy for their primary disease state due to the associated risks of contracting SARS-CoV-2 and the reduction in resources available to healthcare workers [22]. Information on the specific etiology of the cancer is scarce within several SARS-CoV-2 studies. However, lung, breast, gastrointestinal, and hematological cancers (ex. lymphoma) have been reported within COVID-19 cohort studies in the United States [19], Italy [23], and China [18]. Further, cervical cancer patients and patients with other unspecified gynecological malignancies have been reported in these studies [18, 19, 23].

While select literature sources provide glimpses of the oncological paradigms exhibited, the already small patient population assessed dwindles even further when stratified by oncological typing. This is a substantial limitation for assessing mortality risk and providing guidelines for management of COVID-19-positive cancer patients. Along similar lines, very little is known about COVID-19 infection in ovarian cancer patients. At the time of writing, there are 22 PubMed articles about the subject, of which 20 of them discuss potential changes to or challenges faced by cancer clinics to better serve ovarian cancer patients. The remaining two articles discuss a total of three ovarian cancer patients and how their treatment was modified due to the current pandemic [24, 25]. Only two of the three ovarian cancer patients were found to be positive for the novel coronavirus, requiring adjuvant treatment with platelets due to the development of chemotherapy-related thrombocytopenia [24]. The remaining ovarian cancer patient discussed tested negative for SARS-CoV-2 infection, but was presumed to be positive based upon patient symptoms and clinical findings (ex. abnormal CT scan findings consistent with pneumonia in COVID-19 patients) [25]. This patient’s cancer regiment was delayed until the resolution of the presenting atypical pneumonia, but otherwise did not receive any adjuvant therapy [25]. Currently, there are no globally accepted guidelines to address cancer patient management in the settings of a pandemic due to a lacking of data available [26]. Recently, an international collaboration has proposed a series of practical approaches for the diagnosis and treatment of cancer patients [26]. However, until more information or an effective therapeutic regimen against SARS-CoV-2 become available, cancer patients will continue to remain at a very high-risk of mortality due to the COVID-19 epidemic [26].

### Withaferin a as a prospective treatment

Withaferin A (WFA) is a steroidal lactone isolated from the plant *Withania somnifera*, also known as Ashwagandha [27]. It is known for its anti-inflammatory properties, as well as its anti-tumorigenic properties [28–30]. Recent work has demonstrated that COVID-19 infections have a large immune component and can result in the development of cytokine storm, a potentially life-threatening immune reaction in which the body release too many cytokines into the blood at a rapid rate [31]. Work from our lab has demonstrated that WFA is capable of reducing the secretion of various proinflammatory cytokines (ex. TNFα, IL-6, IL-8, and IL-18) in a metastatic model of ovarian cancer [30]. It is within the realm of possibility that WFA treatment can abrogate the intensity of cytokine storm due to the reported anti-inflammatory properties. Interestingly, at least three independent research groups have suggested that phytochemicals found in the plant *Withania somnifera* could be developed as a therapeutic agent against COVID-19 infection using molecular docking approaches [32–34]. Two of the groups reported that various Withanolides, such as WFA, should be able to bind to the viral S-protein receptor binding domain, thereby blocking or reducing interactions with host ACE2 receptor [32, 33]. The third group reported that WFA and a separate withanolide, Withanone, are predicted to interact with the main protease of SARS-CoV-2, although WFA is predicted to have less of a binding affinity than an established N3 protease inhibitor used for baseline docking scores [34].

In an unrelated study, our group has been investigating WFA as a potential therapeutic to treat cancer, including the targeting of cancer stem cells and cancer-induced cachexia (a muscle wasting disorder). As Ang-II signaling is a known mediator of skeletal muscle atrophy [35], we investigated the effect of WFA on Ang-II signaling as it pertains to cachexia. Data (under publication) from our lab has indicated that WFA treatment can reduce circulating levels of Angiotensin II in an experimental model of cancer-induced cachexia. In this study, we xenografted the ovarian cancer cell line A2780 (8.0 × 10^5^ low passage cells resuspended in 100 μl sterile PBS) intraperitoneally into 5 to 6-week old female NOD.Cg-*Prkdc*^*scid*^*Il2rg*^*tm1Wjl*^/SzJ (NSG; Jackson Lab Strain # 005557) mice. Tumor-free controls received an equivalent i.p. injection of sterile saline. After an 8-day of refractory period to allow engraftment of the ovarian cancer cells, tumor-free and tumor-bearing animals received i.p. injections of WFA (2 mg/kg) or vehicle (10% dimethyl sulfoxide, 90% glycerol trioctanoate) once every 3 days over the period of 4 weeks (post-xenografting).

**Fig. 1:**
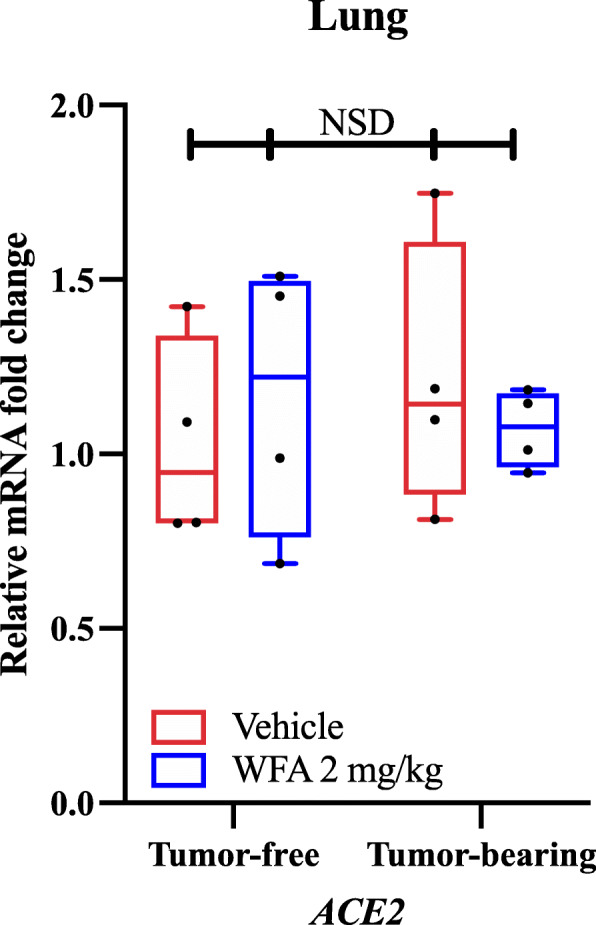
Withaferin A’s effect on ACE2 mRNA expression. (**A**) Relative mRNA levels of *ACE2* in lung samples of tumor-free and A2780 ovarian tumor-bearing female NSG mice treated with vehicle or WFA (2 mg/kg). *N* = 4–5 mice per group. Black circles indicate individual data points. NSD = No significant differences

Using qPCR and gene specific primers, we found that WFA treatment reduced the relative mRNA expression of *AT1R* (Angiotensin II Receptor Type 1) compared to the vehicle-treated group in tumor samples as determined by a two-way analysis of variance (ANOVA) followed by Tukey’s multiple comparison test post hoc analysis. Based upon our findings and the independently reported molecular docking studies, we investigated whether or not WFA treatment would alter *ACE2* expression in the lungs under tumor-free and tumor-bearing conditions. Interestingly, we found no significant differences (NSD; *p*-values > 0.80 for all comparisons) in relative mRNA expression of *ACE2* in response to WFA treatment as determined by a two-way ANOVA (Fig. 1). As we did not observe any significant differences in *ACE2* mRNA expression in the lungs via qPCR, one of the primary regions where ACE2 is expressed, we did not investigate the expression of ACE2 in other organs. However, it was recently reported that, as a byproduct of SARS-CoV-2 infection, ACE2 expression is decreased as part of the disease process, which in turn facilitates the development of multiorgan damage [36]. Due to this effect, others have suggested that blocking the binding of SARS-CoV-2 to the ACE2 receptor may be a more beneficial strategy to combat the virus than augmenting ACE2 expression, due to its antagonistic effect on AT1R signaling [9]. In line with this rationale, it is within the realm of possibility that WFA can block or impede COVID-19 through interactions with the viral S-protein based upon the molecular docking studies [32, 33], without affecting *ACE2* expression (as reported in our data) leading to a worsening of the pathological state.

## Conclusion

The COVID-19 outbreak has become a significant clinical threat worldwide to both the general population and healthcare workers. Additionally, cancer patients and the elderly remain a very high-risk subpopulation that are more susceptible to disease-related fatality. While knowledge about this virus remains limited, over 100 clinical trials are currently being performed to help find a means to combat this epidemic. Due to their potentially immune-compromised position and associated rates of mortality, it would seem that special considerations should be taken in developing a potential therapeutic regimen for cancer patients and patients with other high-risk comorbidities. Withaferin A alone or in combination with drugs, such as: hydroxychloroquine, dexamethasone or other treatments (under clinical trials), could be developed into an attractive therapeutic agent for both the general population and cancer patients due to its anti-tumorigenic properties and the preliminary studies showing that it is capable of binding to the S-protein of SARS-CoV-2, thereby potentially inhibiting infection and/or spread of the disease.

## Data Availability

All data generated or analyzed during this study are included in this published article.

## Abbreviations

ACE2: Angiotensin-converting enzyme-2
Ang: Angiotensin
ANOVA: Analysis of variance
AT1R: Angiotensin II Receptor Type 1
ARDS: Acute respiratory distress syndrome
COVID-19: Coronavirus disease 2019
ICU: Intensive care unit
SARS-CoV-2: Severe acute respiratory syndrome coronavirus-2
NSD: No significant differences
RAS: Renin angiotensin system
S-protein: Spike protein
WFA: Withaferin A
WHO: World Health Organization
